# Comparison of Work Patterns Between Physicians and Advanced Practice Practitioners in Primary Care and Specialty Practice Settings

**DOI:** 10.1001/jamanetworkopen.2023.18061

**Published:** 2023-06-13

**Authors:** Lisa S. Rotenstein, Nate Apathy, Susan Edgman-Levitan, Bruce Landon

**Affiliations:** 1 Department of Medicine, Brigham and Women’s Hospital, Boston, Massachusetts; 2 Leonard Davis Institute of Health Economics, University of Pennsylvania, Philadelphia; 3 Now with MedStar Health Research Institute, Hyattsville, Maryland; 4 Stoeckle Center for Primary Care, Massachusetts General Hospital, Boston; 5 Department of Healthcare Policy, Harvard Medical School, and Beth Israel Deaconess Medical Center, Boston, Massachusetts

## Abstract

**Importance:**

Despite the increasing involvement of advanced practice practitioners (APPs; ie, nurse practitioners and physician assistants) in care delivery across specialties, the work patterns of APPs compared with physicians and how they are integrated into care teams have not been well characterized.

**Objective:**

To characterize differences between physicians and APPs across specialty types related to days with appointments, visit types seen, and time spent using the electronic health record (EHR).

**Design, Setting, and Participants:**

This nationwide, cross-sectional study used EHR data from physicians and APPs (ie, nurse practitioners and physician assistants) at all US institutions that used Epic Systems’ EHR between January and May 2021. Data analysis was performed from March 2022 to April 2023.

**Main Outcomes and Measures:**

Appointment scheduling patterns, percentage of new and established and level of evaluation and management (E/M) visits, and EHR use metrics per day and week.

**Results:**

The sample consisted of 217 924 clinicians across 389 organizations, including 174 939 physicians and 42 985 APPs. Although primary care physicians were more likely than APPs to have more than 3 days per week with appointments (50 921 physicians [79.5%] vs 17 095 APPs [77.9%]), this trend was reversed for medical (38 645 physicians [64.8%] vs 8124 APPs [74.0%]) and surgical (24 155 physicians [47.1%] vs 5198 APPs [51.7%]) specialties. Medical and surgical specialty physicians saw 6.7 and 7.4 percentage points, respectively, more new patient visits than did their APP counterparts, whereas primary care physicians saw 2.8 percentage points fewer new patient visits than did APPs. Physicians saw a greater percentage of level 4 or 5 visits across all specialties. Medical and surgical physicians spent 34.3 and 45.8 fewer minutes per day, respectively, using the EHR than did APPs in their specialties, whereas primary care physicians spent 17.7 minutes per day more. These differences translated to primary care physicians spending 96.3 minutes more per week using the EHR than APPs, whereas medical and surgical physicians spent 149.9 and 140.7 fewer minutes, respectively, than did their APP counterparts.

**Conclusions and Relevance:**

This cross-sectional, national study of clinicians found significant differences in visit and EHR patterns for physicians compared with APPs across specialty types. By underscoring the different current usage of physicians vs APPs across specialty types, this study helps place into context the work and visit patterns of physicians compared with APPs and serves as a foundation for evaluations of clinical outcomes and quality.

## Key Points

**Question:**

How do work patterns of physicians and advanced practice practitioners (APPs; ie, nurse practitioners and physician assistants) vary by specialty?

**Findings:**

In this cross-sectional study of 217 924 clinicians, medical and surgical specialty physicians saw 6.7 and 7.4 percentage points more new patient visits, respectively, than their APP counterparts, whereas primary care physicians saw 2.8 percentage points fewer new patient visits compared with APPs. Medical and surgical physicians spent 34.3 and 45.8 fewer minutes per day, respectively, using the electronic health record than did APPs in their specialties, whereas primary care physicians spent 17.7 more minutes per day than did APPs.

**Meaning:**

These differences in visit and electronic health record patterns for physicians vs APPs by specialty help place into context the work and visit patterns of physicians compared with APPs and serve as a foundation for evaluations of clinical outcomes and quality.

## Teaser

This cross-sectional study examines differences between physicians and advance practice practitioners (APPs) across specialty types related to days with appointments, visit types seen, and time spent on the electronic health record.

## Introduction

Advanced practice practitioners (APPs; ie nurse practitioners and physician assistants) represent an increasing percentage of the US clinician workforce across both primary care and non–primary care specialties.^1,2^ According to US Census data, between 2016 and 2022, the number of persons employed as nurse practitioners and physician assistants increased by 49.4% and 73.9%, respectively, vs a 3.1% decrease in the number of persons employed as physicians.^3,4^ Multiple forces contributed to this growth, including physician shortages, lower labor costs for APPs, increasing demand for care, and evidence regarding the benefits of team-based care, including in some circumstances, care provided by APPs.^5,6^

As the representation of APPs in our health care workforce increases, understanding how they are currently integrated into care delivery and their work and practice patterns is important for contextualizing outcomes of care and further defining their optimal role in our health care system. There are a variety of models for integration of APPs into care delivery. In primary care and in the context of the patient-centered medical home, these range from APPs carrying their own independent panels of patients to APPs taking a larger role in ongoing chronic disease management, leaving physicians to see patients who are more ill and have more complex conditions.^7^ At present, there is less evidence on the role of APPs in provision of specialty care. In medical specialties such as oncology, APPs generally work collaboratively with physicians and see patients in both independent and shared visits,^8^ whereas in surgical specialties such as otolaryngology, they increasingly conduct less-complex, specialty-specific procedures and often perform routine postprocedure follow-up.^9^

Despite the increasing involvement of APPs in care delivery across specialties, the work patterns of APPs compared with physicians and how they are integrated into care teams has not been well characterized. This understanding is crucial for designing effective multidisciplinary teams, particularly in primary care, where APPs have been considered a key part of the movement toward team-based care, as well as a key strategy in alleviating workforce challenges facing primary care.^10,11^ One tool that can provide insights into the ways that APPs are being integrated into care is to examine data that can be extracted from electronic health records (EHRs). Data from EHRs can provide insight into time expenditure during and after hours by physicians and APPs and can potentially highlight opportunities for redistribution of work among role groups to enhance job feasibility. By highlighting the types and complexity of visits seen by each role group, EHR data also can inform prioritization or reconsideration of the types of appointments scheduled for physicians vs APPs to facilitate the best care delivery. We used nationwide, cross-sectional EHR data to explore 3 main issues across outpatient specialty types: How do physicians and APPs vary in terms of days with appointments and EHR activity, types of visits, and quantity and distribution of EHR time?

## Methods

This cross-sectional study leveraged EHR use metadata, including visit (with the term *appointment* used synonymously) and billing information for all US physicians and APPs (ie, nurse practitioners and physician assistants) who used Epic Systems’ EHR from January through April 2021. We derived our primary study measures from weekly, clinician-level measures available through the data warehouse.^3,4,5^ All clinicians and organizations were deidentified before receipt of the data. The University of Pennsylvania institutional review board deemed this study exempt as non–human participants research given the deidentified nature of the data; thus, informed consent was not sought, in accordance with 45 CFR §46. This study followed the Strengthening the Reporting of Observational Studies in Epidemiology (STROBE) reporting guidelines for cross-sectional studies.

### Measures

#### Organization-Level and Clinician-Level Variables

To preserve deidentification of the data, the only organizational level data available through Signal’s data warehouse were organizational structure (ambulatory only, hospital and clinic facilities, and other) and US Census region.^12^ For each of the organizations in our sample, we summed the number of physicians and APPs and calculated the ratio of physicians to APPs. We additionally summed the total number of weekly visits across clinicians to calculate the total weekly number of visits across the organization.

Consistent with previous work^13^ examining differences in the EHR’s measures across outpatient specialty types, physicians and APPs included in our analysis were grouped into primary care, medical specialties, or surgical specialties on the basis of their noted specialty. The eAppendix in Supplement 1 depicts the grouping of individual specialties. We originally classified dermatology and reproductive endocrinology as surgical specialties given their procedural emphasis. However, given that the training of the physicians in these specialties often derives from internal medicine and, thus, key practice features may be more similar to medical specialties, we performed sensitivity analyses where we classified dermatology and reproductive endocrinology as medical rather than surgical specialties. Of note, if uncertainty exists about the specialty categorization of APPs, they are typically assigned as “Other Specialties” in the EHR. Because we could not conduct our analyses without a specialty categorization, we excluded the group of 26 710 clinicians with a categorization of Other Specialties. According to our analyses, APPs comprised 97.5% of these clinicians. However, characteristics of the physician and APP samples with and without these clinicians were similar (eTables 1 and 2 in Supplement 1).

Additional clinician-level variables included mean number of days with appointments per week, percentage of clinicians with a mean of 3 or more days with appointments per week, mean number of appointments per week, and mean days with EHR activity per week. We restricted our analyses to clinicians who had at least 7 weeks of EHR metadata and saw at least 10 patients per week, on average.

#### Visit-Level Information

For each clinician, we also collected the percentage of weekly new and established evaluation and management (E/M) visits and the percentage of weekly E/M visits billed at E/M levels 1 through 5. For the purposes of our analyses, we grouped visit levels into lower complexity (E/M levels 1 and 2), medium complexity (E/M level 3), and higher complexity (E/M levels 4 and 5) categories.

#### Time-Based Measures

We identified weekly total time spent by clinicians using the EHR, as well as time spent after hours, and on the specific activities of notes, Epic’s In Basket feature, and clinical review. We divided each of these measures by the weekly average number of days with appointments to develop clinician-level, per-day averages of time spent in each of these categories. In addition to reporting per-day values, we report weekly totals where appropriate to provide intuitive comparisons across clinicians and specialties that vary in their appointment scheduling. We did not use visits as the denominator for our time-based measures because APPs’ visits are included in the Signal total visit count only if APPs are seeing patients independently; in contrast, APPs’ EHR time is counted whether they are seeing visits independently or in collaboration with a physician.

Of note, the EHR defines active time within the EHR as the time a user is performing active tasks.^14^ If no activity is detected for 5 seconds, the system stops counting time. This measurement allows our data to capture actual EHR work while excluding time a clinician spends with the EHR open but performing other tasks, such as performing a physical examination or speaking with a patient.^15^ These measures may underestimate true EHR work time, because clinicians often spend time reading notes or otherwise performing EHR tasks without directly interacting with the system.^14,16^ In addition, in the database, after hours active time (also known as “Time Outside Scheduled Hours”) is defined as any active time that falls outside a 30-minute buffer before or after the physician’s first and final visits for the day.

### Statistical Analysis

Data analysis was performed from March 2022 to April 2023. We first calculated means and SDs of total physicians, total APPs, physician-to-APP ratios, total weekly visits, and organizational characteristics (region and organization type) for the organizations comprising our sample. Subsequently, we described organizational characteristics and specialty distribution among physicians and APPs and compared these values between the 2 groups using χ^2^ tests. To characterize visit scheduling patterns for physicians vs APPs, we compared mean weekly visits, days with appointments, and days with EHR activity for physicians vs APPs in each specialty category using Kruskal-Wallis tests and percentage of clinicians with 3 or more days with appointments using χ^2^ tests.

To determine how APPs and physicians across specialties differ in the types of visits they see, we compared each clinician type’s mean share of new and established visits and by E/M billing level. To analyze differences in how APPs and physicians spend their time, we calculated per day means of total EHR time, time outside of scheduled hours, daily time working on notes, daily In Basket time, and daily time on clinical review. We compared unadjusted differences in these per-day EHR use metrics for physicians vs APPs by specialty category using Kruskal-Wallis tests. Finally, we examined the extent to which differences in EHR use metrics by clinician type persisted in ordinary least squares regression models adjusting for organization type, region, weekly visits, and percentage of clinicians’ visits at each E/M level. Because per-day values of time-based outcomes may mask some aspects of heterogeneity, we repeated our unadjusted and adjusted analyses using weekly totals of time spent in each domain. All analyses were conducted using SAS OnDemand for Academics version 2021 (SAS Institute, Inc), with a 2-sided significance threshold of *P* < .05.

## Results

### Organization and Clinician Characteristics

Our sample consisted of 217 924 clinicians across 389 organizations, including 174 939 physicians and 42 985 APPs. Among all clinicians, 70 576 (32.4%) were in medical specialties, 85 973 (39.5%) were in primary care specialties, and 61 375 (28.2%) were in surgical specialties. Most of the sample (189 535 clinicians [86.9%]) worked in an organization with both hospital and clinic facilities. More than one-half of APPs (21 954 APPs [51.1%]) were in primary care specialties. Physicians were more equally distributed across specialties, with 64 019 (36.6%) in primary care specialties, 59 597 (34.1%) in medical specialties, and 51 323 (29.3%) in surgical specialties (Table 1). The organizations represented by this sample conducted a mean (SD) of 23 251.8 (22 834.8) visits per week (median [IQR], 16 177.9 [8089.7-31 963.1] visits per week). They had a mean (SD) of 449.7 (459.5) physicians (median [IQR], 295.0 [130.0-612.0] physicians) and 110.5 (143.9) APPs (median [IQR], 67.0 [28.0-138.0] APPs), for a mean physician to APP ratio of 9.6:1.0, with a median (IQR) ratio of 3.8 (2.3-7.5). This ranged from a mean (SD) of 167.2 (177.3) physicians (median [IQR], 110.0 [50.0-228.0] physicians) and 57.3 (83.7) APPs (median [IQR], 31.0 [11.0-74.0] APPs) in primary care (mean ratio, 9.5:1.0) to 154.8 (169.8) physicians (median [IQR], 105.0 [40.0-214.0] physicians) and 28.5 (44.7) APPs (median [IQR], 12.0 [2.0-37.0] APPs) in medical specialties (mean ratio, 15.6:1.0) and 134.7 (142.6) physicians (median [IQR], 69.0 [38.0-182.0] physicians) and 26.4 (37.3) APPs (median [IQR], 14.0 [4.0-34.0] APPs) in surgical specialties (mean ratio, 10.2:1.0). A summary of the characteristics of the organizations in our sample is found in Table 2.

**Table 1.** Demographic and Appointment Characteristics, by Clinician and Specialty Type

Characteristic	Primary care			Medical specialty			Surgical specialty		
	Appointments, No. (%)		*P* value	Appointments, No. (%)		*P* value	Appointments, No. (%)		*P* value
	Physician (n = 64 019)	APP (n = 21 954)		Physician (n = 59 597)	APP (n = 10 979)		Physician (n = 51 323)	APP (n = 10 052)	
Region									
Midwest	18 058 (28.2)	7403 (33.7)	<.001	16 030 (26.9)	3375 (30.7)	<.001	14 294 (27.9)	2810 (28.0)	<.001
Northeast	11 821 (18.5)	4470 (20.4)		14 663 (24.6)	2903 (26.4)		10 359 (20.2)	2781 (27.7)	
South	14 442 (22.6)	5551 (25.3)		14 207 (23.8)	3141 (28.6)		12 682 (24.7)	2556 (25.4)	
West	19 698 (30.8)	4530 (20.6)		14 697 (24.7)	1560 (14.2)		13 988 (27.3)	1905 (19.0)	
Organization type									
Ambulatory only and other	9922 (15.5)	4642 (21.1)	<.001	5925 (10.0)	1264 (11.5)	<.001	5364 (10.5)	1272 (12.7)	<.001
Hospital and clinic facilities	54 097 (84.5)	17 312 (78.9)		53 672 (90.1)	9715 (88.5)		45 959 (90.0)	8780 (87.4)	
Days with appointments per week, mean (SD)	3.8 (0.9)	3.7 (0.9)	<.001	3.5 (1.0)	3.6 (0.9)	<.001	3.0 (1.0)	3.1 (1.1)	<.001
Weekly appointments, mean (SD)	51.1 (27.3)	42.0 (37.6)	<.001	34.0 (24.9)	30.2 (18.9)	<.001	42.2 (26.2)	32.6 (19.2)	<.001
Days with electronic health record activity per week, mean (SD)	5.2 (1.0)	4.6 (1.0)	<.001	5.3 (0.9)	4.7 (0.9)	<.001	5.1 (1.0)	4.6 (0.9)	<.001
>3 d with visits per week	50 921 (79.5)	17 095 (77.9)	<.001	38 645 (64.8)	8124 (74.0)	<.001	24 155 (47.1)	5198 (51.7)	<.001

Abbreviation: APP, advanced practice practitioner.

**Table 2.** Characteristics of Organizations in Sample

Characteristic	Organizations, No. (%) (N = 389)
Region	
Midwest	100 (25.7)
Northeast	73 (18.8)
South	116 (29.8)
West	100 (25.7)
Facility type	
Ambulatory only and other	64 (16.5)
Hospital and clinic facility	325 (83.6)
Total No. weekly visits	
Mean (SD)	23 251.8 (22 834.8)
Median (IQR)	16 177.9 (8089.7-31 963.1)
Total No. of physicians	
Mean (SD)	449.7 (459.5)
Median (IQR)	295.0 (130.0-612.0)
Total No. of APPs	
Mean (SD)	110.5 (143.9)
Median (IQR)	67.0 (28.0-138.0)
Ratio of physicians to APPs	
Mean (SD)	9.6 (19.9)
Median (IQR)	3.8 (2.3-7.5)

Abbreviation: APP, advanced practice practitioner.

### Clinicians’ Visit Scheduling Patterns

Primary care physicians were significantly more likely than APPs to have more than 3 days with appointments per week (50 921 physicians [79.5%] vs 17 095 APPs [77.9%]). This trend was reversed in medical and surgical specialties, where physicians were significantly less likely to have more than 3 days with appointments per week (medical specialties, 38 645 physicians [64.8%] vs 8124 APPs [74.0%]; surgical specialties, 24 155 physicians [47.1%] vs 5198 APPs [51.7%]).

Physicians in all specialties saw more appointments per week than did APPs, with mean (SD) weekly appointments for physicians vs APPs as follows: 51.1 (27.3) vs 42.0 (37.6) for primary care, 34.0 (24.9) vs 30.2 (18.9) for medical specialties, and 42.2 (26.2) vs 32.6 (27.9) for surgical specialties. Physicians in primary care specialties had significantly more (although numerically similar) days with EHR activity per week than did APPs (mean [SD], 3.8 [0.9] vs 3.7 [0.9] days), whereas this trend was reversed for medical specialties (mean [SD], 3.5 [1.0] vs 3.6 [0.9] days) and surgical specialties (mean [SD], 3.0 [1.0] vs 3.1 [1.1] days).

### Clinicians’ Visit Types

**Figure 1.**
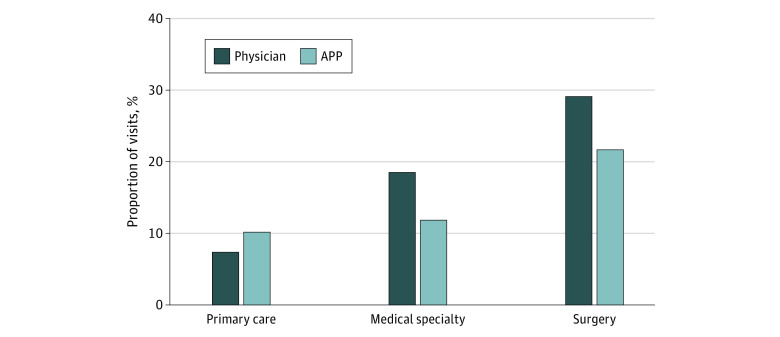
Percentage of All Evaluation and Management Visits Billed as New Visits for Physicians vs Advance Practice Practitioners (APPs)

**Figure 2.**
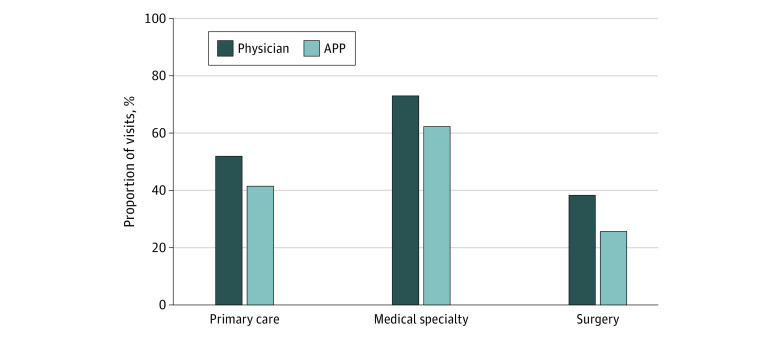
Percentage of All Evaluation and Management Visits Billed at Level 4 or 5 for Physicians vs Advance Practice Practitioners (APPs)

Both medical and surgical specialty physicians saw more new patient visits than APP counterparts (6.7 and 7.4 percentage points more new patient visits, respectively) (Figure 1). In contrast, primary care physicians saw 2.8 percentage points fewer new patient visits than did APP counterparts. Physicians saw a greater percentage of level 4 or 5 visits across all specialties compared with APPs (10.4% more for primary care physicians, 10.7% more for medical physicians, and 12.7% more for surgical physicians) (Figure 2). Absolute visit distribution percentages by specialty and clinician type are displayed in eTables 3 and 4 in Supplement 1.

### Clinicians’ EHR Time

**Figure 3.**
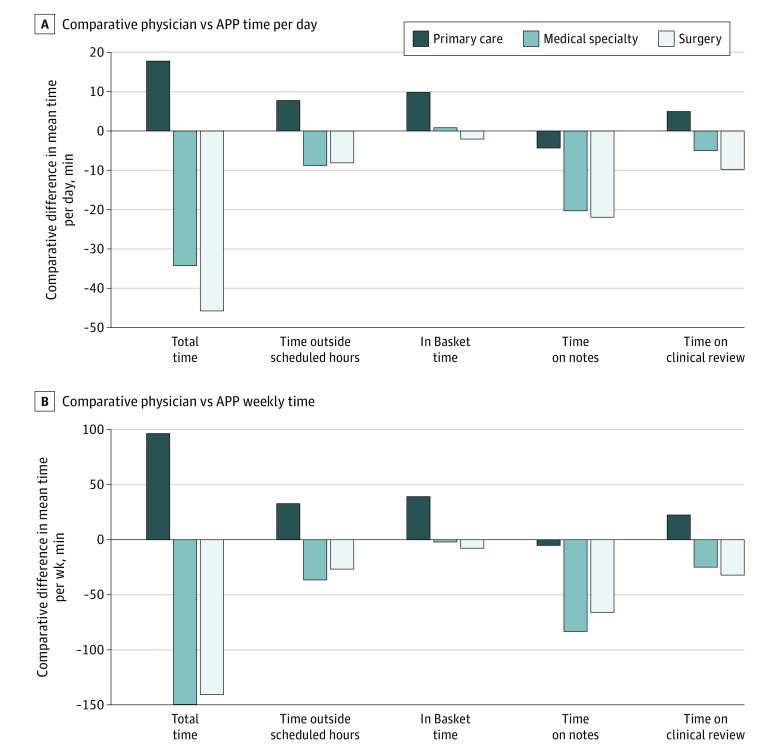
Comparative Difference in per Day and Weekly Electronic Health Record Time Metrics, by Clinician Type and Specialty Category Graphs show comparative physician vs advance practice practitioner (APP) time per day (A) and comparative physician vs APP weekly time (B).

There were significant unadjusted differences in daily EHR time by clinician type and specialty (Figure 3A). Medical and surgical physicians spent 34.3 and 45.8 fewer minutes per day in total, respectively, using the EHR than did APPs in their specialties. In contrast, primary care physicians spent 17.7 minutes more per day than APPs in their specialties. Although medical and surgical physicians spent 8.8 and 8.1 fewer minutes per day, respectively, outside of scheduled hours than APP counterparts, primary care physicians spent 7.8 minutes more per day outside scheduled hours than did primary care APPs.

Surgical physicians spent 2.1 fewer minutes per day using the In Basket than did surgical APPs, whereas medical specialty and primary care physicians spent 0.8 minutes and 9.8 minutes more per day, respectively. Although medical and surgical physicians spent 20.3 and 22.0 fewer minutes per day on notes, respectively, than their APP counterparts, this gap in time on notes per day was only 4.3 minutes per day for primary care physicians vs APPs. All differences were significant and persisted in adjusted analyses (eTable 5 in Supplement 1). Absolute daily EHR time figures by specialty and clinician type are displayed in eTable 6 in Supplement 1.

These differences translated to primary care physicians spending 96.3 minutes (1.6 hours) more per week using the EHR than did APPs, whereas medical and surgical physicians spent 149.9 minutes (2.5 hours) and 140.7 minutes (2.4 hours) less per week, respectively, than did their APP counterparts (Figure 3B). Similarly, primary care physicians spent 32.5 more minutes weekly outside of scheduled hours using the EHR than did APPs, whereas medical and surgical physicians spent 36.8 and 26.9 fewer minutes per week, respectively.

Surgical physicians and medical specialty physicians spent 8.0 minutes and 2.2 fewer minutes per week, respectively, using the In Basket than did surgical APPs, whereas primary care physicians spent 39.5 minutes more weekly than did their APP counterparts. Finally, although medical and surgical physicians spent 83.7 minutes (1.4 hours) and 64.4 minutes (1.1 hours) less per week on notes, respectively, than did their APP counterparts, this gap in time on notes per day was only 5.5 minutes for primary care physicians vs APPs. All differences were significant and persisted in adjusted analyses (eTable 7 in Supplement 1). Absolute weekly EHR time figures by specialty and clinician type are displayed in eTable 8 in Supplement 1. Sensitivity analyses with facility-level fixed effects yielded similar results (eTables 9 and 10 in Supplement 1), as did sensitivity analyses classifying dermatology and reproductive endocrinology as medical specialties.

## Discussion

In this cross-sectional, national study of outpatient clinicians, we leveraged EHR metadata and billing information to demonstrate significant differences in practice and EHR use patterns for physicians vs APPs across primary care, medical, and surgical specialties. Although primary care physicians saw more established visits than did APPs, medical and surgical physicians saw fewer established visits. Medical and surgical physicians spent significantly less total EHR time, time outside scheduled hours, and time on notes than did APPs in their specialties. In contrast, primary care physicians spent more time per week using the EHR in total and outside scheduled hours compared with their APP counterparts, while spending similar time on notes.

The differential distribution of days with appointments and percentage of established visits by role type across specialties suggest different approaches to team-based care for primary care vs medical specialties. Traditionally suggested models of team-based primary care involve physicians seeing new and the most complex cases, while working collaboratively with APPs to care for patients with chronic disease, routine follow-up, and urgent issues.^11^ Although our data suggest a greater presence of this model in medical and surgical specialties, with APPs seeing more established visits and having a greater weekly load of days with appointments, APPs appear to be providing enhanced access to primary care, seeing more new visits than physician counterparts. Notably, this difference is present even though we are unable to quantify instances in which a visit was scheduled under a physician’s name, but was largely conducted by an APP or in conjunction with an APP. Our findings suggest that even as progress has been made toward enhanced functioning of teams within the context of patient-centered medical homes, there has been less emphasis on distinguishing the roles of physicians vs APPs in primary care. APPs appear to serve a more substitutive role in primary care, compared with the complementary role APPs play in medical and surgical specialties.

The EHR time patterns we describe highlight substantially different interactions of physicians and APPs with the EHR and documentation by specialty. In medical and surgical specialties, APPs are taking on more EHR-based activities than their physician counterparts. However, this trend was reversed for primary care physicians and APPs. Over the course of a week, medical and surgical physicians spent 2.5 and 2.4 hours less time, respectively, using the EHR than did APPs in their specialties whereas primary care physicians spent 1.6 hours more each week than did APPs. In addition, although medical and surgical physicians spent 1.4 and 1.1 hours less, respectively, on notes over the course of a week than did their APP counterparts, primary care physicians spent only 5.5 minutes less than did primary care APPs. With surgical and medical physicians spending substantially less time using the EHR, they are potentially freed up for other tasks that they consider of higher value, such as time in the operating room or performing other procedures. In contrast, physicians and APPs in primary care appear to be performing more similar tasks, including bearing a burden of documentation similar to that of APP counterparts. It is notable that physicians across all specialty types did have more days with EHR activity per week than did APP counterparts (although this did not necessarily translate to more absolute EHR time), suggesting that physicians may be expected to interact with the EHR daily even if they do not bear an equivalent burden of its work.

Across all 3 specialties, physicians coded more complex visits than did APPs. This can be explained, in part, by differences in the roles performed by APPs across the different specialties, with surgical and medical specialty APPs less frequently providing comprehensive new visits and more frequently providing follow-up care and lower complexity, routine visits. In addition, it is likely more common for physicians to have incentives to deliver and code higher level visits and see more patients per session. According to a 2019 national survey, only one-half of organizations were providing productivity-based incentives for APPs,^17^ compared with approximately 70% for physician organizations.^18^ For APPs, these incentives are most commonly used in primary care–based specialties and practice settings where APPs practice and bill independently.^17^ A greater understanding of the differential visit complexity coding seen in our study will ultimately be helpful for contextualizing previous mixed evidence about relative outcomes and quality of care for physicians vs APPs^5,6,19,20,21^ and designing optimal teams going forward.

### Limitations and Strengths

This study has several limitations. First, we are unable to differentiate between APPs who work collaboratively with physicians and those who carry their own panels; this limited our ability to accurately normalize our time measures to the visit level rather than the day level. In addition, data were only available aggregated at the clinician week level, without specific visit details such as the clinical diagnoses addressed. Because our data are based on users of the Epic EHR, they may not be representative of practice patterns in smaller practices that are less likely to use Epic. It also is possible that some of the differences in EHR time identified, particularly differences in note time, are associated with differing behavioral patterns around documentation for APPs vs physicians. We were unable to categorize a substantial portion of APPs and physicians whose specialty is defined as other. However, our sample’s demographic characteristics were similar both with and without these clinicians included. Our data did not include specialty training information for APPs; rather, the specialties associated with APPs represent the clinical setting in which they practice and use the EHR. Furthermore, our data derive from a period during which COVID-19 was present, which may have influenced some aspects of care delivered. However, we expect many of the trends associated with this period (eg, increased use of telemedicine) to continue even after the acute phase of the pandemic has passed. These limitations are balanced by several strengths. The data for our study derive from more than 217 000 clinicians across the US across geographies, health systems, and specialties. We were uniquely able to characterize billing and EHR use patterns for these clinicians and also characterize these patterns by specialty.

## Conclusions

In this cross-sectional study, we found significant differences in visit and EHR use patterns for physicians and APPs by specialty type. We also showed that the ways in which APPs are used for visit-based care and EHR-based work, including documentation, differed markedly by specialty. Our findings underscore the different roles of physicians vs APPs across specialty types, and, in particular, highlight role differences in primary care vs other specialties. This study helps place into context the work and visit patterns of physicians compared with APPs and serves as a foundation for evaluations of clinical outcomes and quality.

## Notes

Supplement 1. **eAppendix.** Specialty Categorizations **eTable 1.** Characteristics of Physician and APP Samples, With and Without “Other Specialties”: Provider-Level Comparisons **eTable 2.** Characteristics of Physician and APP Samples, With and Without “Other Specialties”: Organization-Level Comparisons **eTable 3.** Mean Percent of New and Established Visits for Physicians Versus APPs, by Specialty **eTable 4.** Mean Percent of Visits at Each E/M Level for Physicians Versus APPs, by Specialty **eTable 5.** Mean Absolute EHR Time Per Day in Minutes for Physicians Versus APPs, by Specialty **eTable 6.** Mean Absolute Weekly Time Per Day in Minutes for Physicians Versus APPs, by Specialty **eTable 7.** Adjusted* Comparative EHR Time Per Day in Minutes for Physicians Versus APPs, by Specialty **eTable 8.** Adjusted* Comparative Weekly EHR Time in Minutes for Physicians Versus APPs, by Specialty **eTable 9.** Adjusted* Comparative EHR Time Per Day in Minutes for Physicians Versus APPs, by Specialty and Including Facility Fixed Effects **eTable 10.** Adjusted* Comparative Weekly EHR Time in Minutes for Physicians Versus APPs, by Specialty and Including Facility Fixed Effects

Supplement 2.
Data Sharing Statement
