# Errors in Methods, Results, Table 1, and Supplement 1

**DOI:** 10.1001/jamanetworkopen.2023.25921

**Published:** 2023-07-13

In the Original Investigation titled “Comparison of Work Patterns Between Physicians and Advanced Practice Practitioners in Primary Care and Specialty Practice Settings,”^1^ published June 13, 2023, there were errors in several numbers in the Methods, Results, Table 1, and Supplement 1. The conclusions were not affected by these corrections. This article has been corrected.^1^
