# Supplementary material for: Comparison of Work Patterns Between Physicians and Advanced Practice Practitioners in Primary Care and Specialty Practice Settings
Source: JAMA Netw Open. 2023 Jun 13;6(6):e2318061. doi: 10.1001/jamanetworkopen.2023.18061 (PMC10265293; doi:10.1001/jamanetworkopen.2023.18061)
Supplement: Supplement 1. — eAppendix. Specialty Categorizations eTable 1. Characteristics of Physician and APP Samples, With and Without “Other Specialties”: Provider-Level Comparisons eTable 2. Characteristics of Physician and APP Samples, With and Without “Other Specialties”: Organization-Level Comparisons eTable 3. Mean Percent of New and Established Visits for Physicians Versus APPs, by Specialty eTable 4. Mean Percent of Visits at Each E/M Level for Physicians Versus APPs, by Specialty eTable 5. Mean Absolute EHR Time Per Day in Minutes for Physicians Versus APPs, by Specialty eTable 6. Mean Absolute Weekly Time Per Day in Minutes for Physicians Versus APPs, by Specialty eTable 7. Adjusted* Comparative EHR Time Per Day in Minutes for Physicians Versus APPs, by Specialty eTable 8. Adjusted* Comparative Weekly EHR Time in Minutes for Physicians Versus APPs, by Specialty eTable 9. Adjusted* Comparative EHR Time Per Day in Minutes for Physicians Versus APPs, by Specialty and Including Facility Fixed Effects eTable 10. Adjusted* Comparative Weekly EHR Time in Minutes for Physicians Versus APPs, by Specialty and Including Facility Fixed Effects [file jamanetwopen-e2318061-s001.pdf]

Supplemental Online Content

Rotenstein LS, Apathy N, Edgman-Levitan S, Landon B. Comparison of work patterns between physicians and advanced practice practitioners in primary care and specialty practice settings. *JAMA Netw Open*. 2023;6(6):e2318061. doi:10.1001/jamanetworkopen.2023.18061

eAppendix. Specialty Categorizations
eTable 1. Characteristics of Physician and APP Samples, With and Without “Other Specialties”: Provider-Level Comparisons
eTable 2. Characteristics of Physician and APP Samples, With and Without “Other Specialties”: Organization-Level Comparisons
eTable 3. Mean Percent of New and Established Visits for Physicians Versus APPs, by Specialty
eTable 4. Mean Percent of Visits at Each E/M Level for Physicians Versus APPs, by Specialty
eTable 5. Mean Absolute EHR Time Per Day in Minutes for Physicians Versus APPs, by Specialty
eTable 6. Mean Absolute Weekly Time Per Day in Minutes for Physicians Versus APPs, by Specialty
eTable 7. Adjusted* Comparative EHR Time Per Day in Minutes for Physicians Versus APPs, by Specialty
eTable 8. Adjusted* Comparative Weekly EHR Time in Minutes for Physicians Versus APPs, by Specialty
eTable 9. Adjusted* Comparative EHR Time Per Day in Minutes for Physicians Versus APPs, by Specialty and Including Facility Fixed Effects
eTable 10. Adjusted* Comparative Weekly EHR Time in Minutes for Physicians Versus APPs, by Specialty and Including Facility Fixed Effects

This supplemental material has been provided by the authors to give readers additional information about their work.

© 2023 Rotenstein LS et al. *JAMA Network Open.*

eAppendix. Specialty Categorizations

Primary Care
Family Medicine
General Internal Medicine
General Pediatrics
Geriatric Medicine
Women's Health

Medical Specialty
Allergy and Immunology
Alternative Medicine
Cardiology
Endocrinology
Gastroenterology
Genetics
Hematology
Infectious Disease
Nephrology
Neurology
Occupational Medicine
Oncology
Pain Medicine
Palliative Care
Physical Medicine
Psychiatry
Pulmonology
Rheumatology
Sleep Medicine
Sports Medicine
Urgent Care

© 2023 Rotenstein LS et al. *JAMA Network Open.*

Surgical Specialty
Cardiothoracic Surgery
Colorectal Surgery
Dermatology
General Surgery
Neurosurgery
Obstetrics and Gynecology
Ophthalmology
Orthopedics
Otorhinolaryngology
Plastic Surgery
Podiatry
Reproductive Endocrinology
Reproductive and Endocrine Surgery
Transplant Surgery
Trauma Surgery
Urology
Vascular Surgery
Wound Medicine

© 2023 Rotenstein LS et al. *JAMA Network Open*.

eTable 1. Characteristics of Physician and APP Samples, With and Without "Other Specialties": Provider-Level Comparisons

	"Other Specialties" Not Included (Main Analysis)		"Other Specialties" Included	
**Characteristic**	**Physician** ***n* = 174,939** **N (%)**	**APP** ***n = 42,985*** **N (%)**	**Physician** ***n* = 175,638** **N (%)**	**APP** ***n = 69,695*** **N (%)**
**Region**				
Midwest	48,382 (27.7)	13,588 (31.6)	48,504 (27.6)	21,111 (30.3)
Northeast	36,843 (21.1)	10,154 (23.6)	36,933 (21.0)	17,028 (24.4)
South	41,331 (23.6)	11,248 (26.2)	41,415 (23.6)	17,100 (24.5)
West	48,383 (27.7)	7,995 (18.6)	48,786 (27.8)	14,456 (20.7)
**Organization Type**				
Ambulatory Only & Other	153,728 (87.9)	35,807 (83.3)	154,388 (87.9)	56,331 (80.8)
Hospital and Clinic Facilities	21,211 (12.1)	7,178 (16.7)	21,250 (12.1)	13,364 (19.2)
	**Mean (SD)**	**Mean (SD)**	**Mean (SD)**	**Mean (SD)**
**Mean Days with Appointments Per Week**	3.5 (1.0)	3.5 (0.9)	3.5 (1.0)	3.5 (0.9)
**Mean Weekly Appointments**	42.7 (27.2)	36.8 (30.5)	42.6 (27.1)	46.6 (85.0)
**Mean Days with EHR Activity Per Week**	5.2 (1.0)	4.6 (1.0)	5.2 (1.0)	4.5 (1.0)
**> 3 Days with Visits Per Week (N (%))**	61,218 (65.0)	30,417 (70.8)	114,176 (65.0)	20,433 (70.7)

© 2023 Rotenstein LS et al. *JAMA Network Open.*

eTable 2. Characteristics of Physician and APP Samples, With and Without "Other Specialties": Organization-Level Comparisons

Characteristic	"Other Specialties" Not Included (Main Analysis) N = 389		"Other Specialties" Included N=389	
	N (%)		N (%)	
**Region**				
Midwest	100 (25.7)		100 (25.7)	
Northeast	73 (18.8)		73 (18.8)	
South	116 (29.8)		116 (29.8)	
West	100 (25.7)		100 (25.7)	
**Customer Type**				
Ambulatory Only	64 (16.5)		64 (16.5)	
Hospital and Clinic Facility Other	325 (83.6)		325 (83.6)	
	**Mean (SD)**	**Median (IQR)**	**Mean (SD)**	**Median (IQR)**
**Total Weekly Visits**	23,251.8 (22,834.8)	16,177.9 (8,089.7, 31,963.1)	27,596.4 (46,289.5)	17,726.85 (8,534.9, 33,921.7)
**Total Number of Physicians**	449.7 (459.5)	295.0 (130.0, 612.0)	451.5 (461.2)	295.0 (130.0, 613.0)
**Total Number of APPs**	110.5 (143.9)	67.0 (28.0, 138.0)	179.2 (253.9)	105.0 (53.0, 229.0)
**Ratio of Physicians to APPs**	9.6 (19.9)	3.8 (2.3, 7.5)	3.5 (3.3)	2.5 (1.8, 3.9)

© 2023 Rotenstein LS et al. *JAMA Network Open.*

eTable 3. Mean Percent of New and Established Visits for Physicians Versus APPs, by Specialty

Specialty	New Visit		Established Visit	
	Physician Mean (SD)	APP Mean (SD)	Physician Mean (SD)	APP Mean (SD)
Primary Care	92.6 (10.8)	10.2 (15.4)	7.4 (10.8)	89.8 (15.4)
Medical Specialty	18.5 (17.5)	11.8 (18.9)	7.4 (10.8)	88.2 (18.9)
Surgery	29.1 (19.9)	21.7 (19.6)	70.9 (19.9)	78.3 (19.6)

eTable 4. Mean Percent of Visits at Each E/M Level for Physicians Versus APPs, by Specialty

Specialty	Level 1 or 2		Level 3		Level 4 or 5	
	Physician Mean (SD)	APP Mean (SD)	Physician Mean (SD)	APP Mean (SD)	Physician Mean (SD)	APP Mean (SD)
Primary Care	6.8 (13.6)	9.3 (17.3)	41.3 (24.1)	49.3 (26.1)	51.9 (27.7)	41.5 (28.9)
Medical Specialty	4.6 (13.1)	6.4 (16.4)	22.4 (23.3)	31.3 (28.5)	73.0 (27.6)	62.3 (32.7)
Surgery	15.0 (21.8)	19.1 (24.8)	46.7 (26.3)	55.3 (27.6)	38.3 (29.4)	25.6 (26.7)

© 2023 Rotenstein LS et al. *JAMA Network Open.*

eTable 5. Mean Absolute EHR Time Per Day in Minutes for Physicians Versus APPs, by Specialty

Specialty	Total EHR Time (in minutes) Mean (SD)		EHR Time Outside Scheduled Hours (in minutes) Mean (SD)		In Basket Time (in minutes) Mean (SD)		Time on Notes (in minutes) Mean (SD)		Time on Clinical Review (in minutes) Mean (SD)	
	Physician	APP	Physician	APP	Physician	APP	Physician	APP	Physician	APP
Primary Care	240.2 (98.2)	222.5 (86.9)	41.4 (35.2)	33.6 (31.1)	34.1 (22.7)	24.3 (18.4)	51.8 (37.0)	89.9 (44.6)	24.4 (16.2)	34.0 (18.5)
Medical Specialty	186.4 (92.9)	220.7 (88.4)	36.7 (30.3)	45.4 (36.6)	22.6 (18.8)	21.8 (19.6)	75.8 (50.2)	96.2 (51.4)	36.5 (23.0)	41.5 (24.1)
Surgery	140.5 (75.2)	186.3 (86.1)	24.1 (21.2)	32.2 (28.4)	18.1 (15.5)	20.2 (17.2)	51.8 (37.0)	73.8 (42.7)	24.4 (16.2)	34.2 (20.4)

eTable 6. Mean Absolute Weekly Time Per Day in Minutes for Physicians Versus APPs, by Specialty

Specialty	Total EHR Time (in minutes) Mean (SD)		EHR Time Outside Scheduled Hours (in minutes) Mean (SD)		In Basket Time (in minutes) Mean (SD)		Time on Notes (in minutes) Mean (SD)		Time on Clinical Review (in minutes) Mean (SD)	
	Physician	APP	Physician	APP	Physician	APP	Physician	APP	Physician	APP
Primary Care	918.0 (424.2)	821.6 (360.7)	159.5 (146.0)	127.0 (125.4)	130.4 (88.2)	91.0 (70.2)	325.0 (185.6)	330.5 (175.7)	148.6 (88.5)	126.2 (73.6)
Medical Specialty	630.7 (350.6)	780.7 (330.2)	127.7 (117.6)	164.5 (140.6)	74.6 (60.7)	76.8 (65.3)	259.2 (189.3)	342.9 (197.9)	122.2 (79.1)	147.4 (85.9)
Surgery	416.4 (259.8)	557.1 (279.1)	73.5 (74.9)	100.4 (99.2)	51.9 (45.7)	59.9 (50.8)	155.9 (126.0)	222.3 (140.5)	71.0 (50.2)	103.0 (64.7)

© 2023 Rotenstein LS et al. *JAMA Network Open.*

eTable 7. Adjusted* Comparative EHR Time Per Day in Minutes for Physicians Versus APPs, by Specialty

Specialty	Total EHR Time	EHR Time Outside Scheduled Hours	In Basket Time	Time on Notes	Time on Clinical Review
**Primary Care**	2.8 (1.3, 4.3) **	7.3 (6.8, 7.9) **	7.0 (6.7, 7.4) **	-8.7 (-9.4, -8.0) **	2.7 (2.4, 3.0) **
**Medical Specialty**	-42.3 (-44.1, -40.4) **	-10.5 (-11.2, -9.9) **	-0.2 (-0.6, 0.2)	-25.0 (-26.1, -24.0) **	-6.1 (-6.6, -5.6) **
**Surgery**	-52.3 (-53.9, -50.6) **	-9.2 (-9.7, -8.7) **	-2.1 (-2.5, -1.8) **	-25.9 (-26.8, -25.1) **	-9.8 (-10.2, -9.4) **

*Comparative time for physicians vs. APPs in each category derived from separate multivariable models with EHR time metric as dependent variable. Models adjust for organization type, region, total weekly visits, and percent of visits at each E/M level.
** Significant at p<0.001 level

eTable 8. Adjusted* Comparative Weekly EHR Time in Minutes for Physicians Versus APPs, by Specialty

Specialty	Total EHR Time	EHR Time Outside Scheduled Hours	In Basket Time	Time on Notes	Time on Clinical Review
**Primary Care**	7.6 (1.7, 13.6) **	26.3 (24.1, 28.5) **	24.9 (23.6, 26.2) **	-34.1 (-36.9, -31.3) **	8.9 (7.6, 10.2) **
**Medical Specialty**	-200.3 (-206.9, -193.7) **	-46.6 (-49.1, -44.1) **	-8.1 (-9.3, -6.8) **	-108.9 (-112.7, -105.2) **	-34.0 (-35.6, -32.4) **
**Surgery**	-200.4 (-205.6, -195.3) **	-36.7 (-38.4, -35.0) **	-10.7 (11.7, -9.7) **	-90.4 (-93.0, -87.8) **	-38.9 (-40.0, -37.7) **

*Comparative time for physicians vs. APPs in each category derived from separate multivariable models with EHR time metric as dependent variable. Models adjust for organization type, region, total weekly visits, and percent of visits at each E/M level.
** Significant at p<0.001 level
*** Significant at p = 0.01 level

© 2023 Rotenstein LS et al. *JAMA Network Open.*

eTable 9. Adjusted* Comparative EHR Time Per Day in Minutes for Physicians Versus APPs, by Specialty and Including Facility Fixed Effects

Specialty	Total EHR Time	EHR Time Outside Scheduled Hours	In Basket Time	Time on Notes	Time on Clinical Review
**Primary Care**	2.0 (0.6, 3.5) ***	7.2 (6.7, 7.7) **	6.8 (6.5, 7.1) **	-8.7 (-9.4, -8.0) **	2.7 (2.4, 3.0) **
**Medical Specialty**	-43.5 (-45.4, -41.7) **	-10.7 (-11.3, -10.1) **	-2.3 (-2.7, -2.0) **	-25.7 (-26.7, -24.6) **	-6.3 (-6.8, -5.8) **
**Surgery**	-53.0 (-54.7, -51.3) **	-9.2 (-9.7, -8.7) **	-0.2 (-0.6, 0.2)	-26.2 (-27.1, -25.4) **	-10.0 (-10.4, -9.6) **

*****Comparative time for physicians vs. APPs in each category derived from separate multivariable models with EHR time metric as dependent variable. Models adjust for organization type, region, total weekly visits, percent of visits at each E/M level, and facility.
** Significant at p<0.001 level
*** Significant at p = 0.01 level

eTable 10. Adjusted* Comparative Weekly EHR Time in Minutes for Physicians Versus APPs, by Specialty and Including Facility Fixed Effects

Specialty	Total EHR Time	EHR Time Outside Scheduled Hours	In Basket Time	Time on Notes	Time on Clinical Review
**Primary Care**	5.2 (-0.7, 11.2)	25.9 (23.7, 28.1) **	24.1 (22.8, 25.4) **	-34.8 (-37.6, -32.0) **	8.0 (6.7, 9.3) **
**Medical Specialty**	-204.9 (-211.5, -198.4) **	-47.4 (-49.9, -44.8) **	-9.2 (-10.4, -8.0) **	-111.2 (-115.0, -107.4) **	-35.1 (-36.6, -33.5) **
**Surgery**	-200.4 (-205.6, -195.3) **	-36.8 (-38.5, -35.1) **	-11.5 (-12.5, -10.5) **	-91.4 (-94.0, -88.8) **	-39.5 (-40.7, -38.4) **

*****Comparative time for physicians vs. APPs in each category derived from separate multivariable models with EHR time metric as dependent variable. Models adjust for organization type, region, total weekly visits, percent of visits at each E/M level, and facility.
** Significant at p<0.001 level

© 2023 Rotenstein LS et al. *JAMA Network Open.*
