# Supplementary material for: Comparison of Work Patterns Between Physicians and Advanced Practice Practitioners in Primary Care and Specialty Practice Settings
Source: JAMA Netw Open. 2023 Jun 13;6(6):e2318061. doi: 10.1001/jamanetworkopen.2023.18061 (PMC10265293; doi:10.1001/jamanetworkopen.2023.18061)
Supplement: Supplement 2. — Data Sharing Statement [file jamanetwopen-e2318061-s002.pdf]

Data Sharing Statement

Rotenstein. Comparison of Work Patterns Between Physicians and Advanced Practice Practitioners in Primary Care and Specialty Practice Settings. *JAMA Netw Open*. Published June 13, 2023. doi:10.1001/jamanetworkopen.2023.18061

Data
Data available: No

Additional Information
Explanation for why data not available: Data shared with authors via a partnership with Epic Systems.
